# Case Report: Primary Immunodeficiencies, Massive EBV+ T-Cell Lympoproliferation Leading to the Diagnosis of ICF2 Syndrome

**DOI:** 10.3389/fimmu.2021.654167

**Published:** 2021-04-28

**Authors:** Gonçalo Luzes Padeira, Catarina Araújo, Ana Isabel Cordeiro, João Freixo, Catarina Gregório Martins, João Farela Neves

**Affiliations:** ^1^ Hospital de Dona Estefânia, Centro Hospitalar Universitário de Lisboa Central, Lisbon, Portugal; ^2^ Departamento de Anatomia Patológica, Centro Hospitalar Universitário de Lisboa Central, Lisbon, Portugal; ^3^ Centro de Genética Preditiva e Preventiva, Instituto de Biologia Molecular e Celular, Instituto de Investigação e Inovação em Saúde, Porto, Portugal; ^4^ CEDOC, Chronic Diseases Research Center, NOVA Medical School, Lisbon, Portugal; ^5^ Comprehensive Health Research Centre (CHRC), NOVA Medical School, Nova University of Lisbon, Lisbon, Portugal

**Keywords:** case report, ICF-2, Primary immune deficiencies, EBV, lymphoproliferation

## Abstract

In immunocompromised patients, EBV may elicit B-cell transformation and proliferation. A 5-year-old microcephalic boy was admitted with fever and non-malignant polymorphic T-cell lymphoproliferative disease associated with EBV. A presumptive diagnosis of primary immunodeficiency with inability to control EBV was made and next-generation sequencing led to the identification of a novel ZBTB24 mutation (ICF2-syndrome). This case shows that susceptibility to EBV seems to be particular of ICF-2 as it has not been described in the other types of ICF. It is mandatory to raise the hypothesis of an underlying PID in case of severe EBV infection.

## Introduction

Epstein-Barr virus (EBV) is one of eight human herpesviruses that establishes lifelong persistent infection in humans. More than 90% of adults have been infected with EBV, whose life cycle mimics the natural differentiation pathway of B cells, giving the virus access to its site of latent infection, the resting memory B cells (1).

Primary infection in early childhood is generally asymptomatic but later in life it usually causes infectious mononucleosis. In some patients, EBV can induce lymphoproliferation, lymphoma, and hemophagocytic lymphohistiocytosis (HLH) particularly in immunocompromised patients, reflecting the importance of continuous immune surveillance for the regulation of the virus (2). In healthy patients the propensity of EBV to induce B-cell proliferation seems to be counterbalanced by an immune system that maintains the overall number of EBV-infected B lymphocytes in a steady level (2).

After infecting B-cells (and epithelial cells), the EBV replicative cycle that follows results in the expression of lytic proteins involved in immune evasion which interfere with antigen (Ag) processing and presentation to CD8^+^ T cells. Then, EBV switches to latent infection of B cells avoiding T-cell and NK-cell immunity, which is critical for colonization of the host. Although the exact mechanisms are still being discussed latency can take several forms (2), starting as a growth-transforming latent infection of B cells where expression of all latent proteins (EBV nuclear Ags (EBNA) 1, 2, 3A, 3B, 3C, and LP; latent membrane proteins (LMPs) 1 and 2) can be detected. In this state infected B cells expand rapidly in extrafollicular areas of oropharyngeal lymphoid tissues such as the tonsils, and large numbers of infected B cells can be found in the blood (latency III). Most of these infected cells are cleared by the immune system (2). Other latency forms consist of latency II which is a more restrict form of latency design to evade the immune system and where only EBNA1 and LMPs are expressed. Finally, only EBNA1 is expressed in latency I. In fact, viral persistence is achieved largely through silent infection of memory B cells where expression of viral Ags is extinguished (latency 0); in healthy carriers, EBV is exclusively found within this population in the blood (2).

Primary immunodeficiencies that present with EBV-driven LPDs are related to defects in the growth, differentiation and activities of B and/or T cells. Briefly, the host defense to EBV can be compromised by germline mutations in genes that encode proteins that are crucial for an adequate response (3): a) in the initial interaction between EBV-infected or antigen-presenting B cells and CD8+ T cells (CD27/CD70, 4-1BB/4-1BBL) and downstream signaling (requiring MAGT1, ITK, and RasGRP1) that will induce DNA synthesis by activation of CTPS1 and elicit proliferation of EBV-specific CD81 T cells; b) or following expansion of EBV-specific CD81 T cells by compromising cytotoxicity of CD8+ T and NK cells as seen in patients with mutations in the SAP pathway. SAP links SLAM-family receptors on hematopoietic cells with downstream intracellular signaling pathways to regulate T and NK cells. In the absence of SAP, cytotoxic functions of CD81 T and NK cells induced by engaging the SLAM-family receptors 2B4 (CD244) and NTB-A are abolished. These two pathways are required for the functioning of one-another as illustrated by the ASGRP1-dependent induction of CTPS1, the impaired NKG2D and 2B4 expression secondary to CD27/CD70 deficiency or by the impaired PLCγ1 activation due to ITK or MAGT1 deficiency (3).

Moreover, in immunocompromised patients who present an impaired defense against EBV, the virus may elicit cell transformation and proliferation, leading to EBV-induced lymphoproliferative disorders (EBV-LPDs) (2). These are usually divided in reactive proliferations (including reactive lesions with no malignant potential like infectious mononucleosis and reactive lesions with varied malignant potential), B cell proliferations (including Hodgkin lymphoma and plasma cell neoplasms), T/natural killer (NK)-cell proliferations and immunodeficiency-related lymphoid proliferations. Combined T-cell and B-cell immunodeficiencies account for about two thirds of PIDs associated with EBV-driven lymphoproliferative disorders (4).

There are several primary immunodeficiencies in which EBV+ B-cell LPDs are an important feature, but very few germline mutations have been associated to documented EBV+ T/NK-cell LPD (4). We report the case of a boy with massive EBV+ T-cell LPD caused by a novel homozygous *ZTBZ24* mutation.

## Case Report

**Figure 1:**
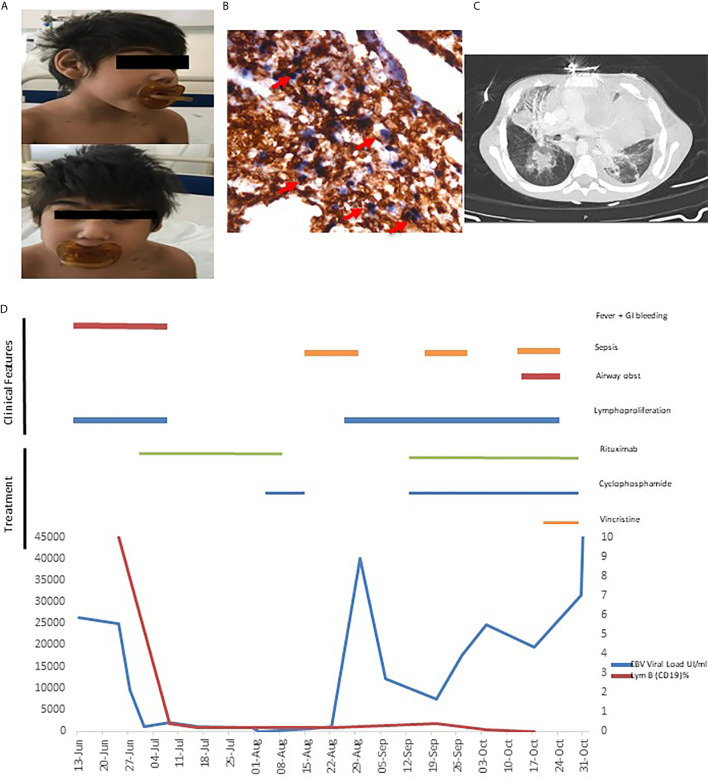
**(A)** Microcephaly **(B)** Gastric biopsy: EBV-encoded small ribonucleic acid (EBER1) in situ hybridization shows positive nuclei EBER+ in the T cells lymphocytes. Double staining EBER-ISH (blue) and CD3-IHC (brown) **(C)** Thorax CT with mediastinal infiltration. **(D)** Clinical evolution, EBV load and therapeutics.

Five-year-old boy with no relevant family background but with personal history of epilepsy, severe psychomotor retardation, microcephaly, micrognathia, hypertelorism, low-set ears, epicanthal folds, and macroglossia (**Figure 1A**).

Admitted in the context of prolonged fever and generalized lymphadenopathies (cervical, abdominal, and mediastinal). He had no other complaints.

An extensive investigation was performed and an elevated EBV viral load (50,000 copies/ml) despite a negative EBV serology was found. Prior to receiving any immunosuppressive treatment, immunologic evaluation was performed and revealed normal serum levels of IgG, IgA and IgM, normal responses to diphteria and tetanus and a severe T and NK-cell lymphopenia, as well as B-cell lymphopenia, particularly low post-germinal class-switched memory B cells (**Table 1**).

**Table 1:** Investigation.

	Reference	
**Infectious**		
**EBV-VCA IgM/IgG** **EBV-EA IgG** **EBV-EBNA IgG**	–	Negative/Negative Negative Negative
**CMV, HSV 1,HSV2, VZV, HHV6, HHV7 PCR**	–	Negative
**CMV IgM/IgG**	–	Negative/**Positive**
**Enterovirus PCR in stools; Multiplex PCR for respiratory virus**	–	Negative
**Parvovirus IgG/IgM**	–	Negative
**HIV 1 and 2**	–	Negative
**VHA IgM/IgG**	–	Negative/**Positive**
**Serology for Coxiella burnetti, Leishmania, Leptospira, Mycoplasma, Toxoplasma**	–	Negative
**IGRA**	–	Negative
**Immunologic**		
**Immunoglobulin G (mg/dL)**	593-1730	903
**Immunoglobulin A (mg/dL)**	33-360	202
**Immunoglobulin M (mg/dL)**	55-210	83
**Immunoglobulin E (KUI/L)**	0-90	10.2
**Lymphocyte populations** **Abs value(cel/uL)** ** Lym B (CD19+ %)** ** Lym B [CD19+ (cells/uL)]** ** PreGerminal %** ** PreGerminal(cells/uL)** ** PostGerminal %** ** PostGerminal(cells/uL)** ** Unswitched %** ** Unswitched(cells/uL)** ** Switched %** ** Switched(cells/uL)** ** Lym T (CD3+ %)** ** Lym T [CD3+ cells/uL)]** ** CD3+/CD4+ %** ** CD3+/CD4+ (cells/uL)** ** Naive %** ** Naive(cells/uL)** ** Central Memory %** ** Central Memory(cells/uL)** ** Effector Memory %** ** Effector Memory(cells/uL)** ** CD3+/CD8+ %** ** CD3+/CD8+ (cells/uL)** ** Naive %** ** Naive(cells/uL)** ** Central Memory %** ** Central Memory(cells/uL)** ** Effector Memory %** ** Effector Memory(cells/uL)** ** Effector TD CD27+ %** ** Effector TD CD27+(cells/uL)** ** Effector TD CD27- %** ** Effector TD CD27-(cells/uL)** ** T TCRgd+ %** ** T TCRgd+(cells/uL)** ** T TCRgd- %** ** T TCRgd-(cells/uL)** **NK CD3-/CD16 56+ %** **NK CD3-/CD16 56+ (cells/uL)**	1.200-6850 6,1-25,2 157-1637 48-88 70-1350 8-33 35-490 3-22 18-333 2-17,4 14-200 55-97 850-5300 26-61 515-3500 42-100 265-2900 1-100 150-800 0.2-18 9-222 13-47 188-1800 16-100 94-1130 1-39 26-450 0,4-100 0-151 0-3,8 0-385 0,01-7,2 1-735 2-24 44-748 0,04-1,3 3-104 2-31 106-1759	** 550** 10,58 **58,2** **96,56** **56,2** **3,44** **2** **0,19** **0,11** 3,26 **1,9** 89 **490** **71,27** **390** **26,28** **103** 71,43 280 2,4 9,4 13,8 **76,1** 46,39 **35,3** **42,05** 32 1,45 1 0,17 **16,5** 0,06 2,2 3,37 16,5 0,17 5,5 0,27 **1,5**

In bold values outside the reference range.

An excisional biopsy of a lymphadenopathy was suggestive of non-malignant polymorphic EBV (EBER+) T-cell lymphoproliferative disease. This lymphoproliferation was accompanied by infiltration of the digestive tract and he developed severe upper and lower digestive bleeding with two episodes of hemorrhagic shock, needing multiple blood transfusions. A gastric ulcer biopsy confirmed a polymorphic T infiltrate (EBER+) (**Figure 1B**) and rearrangements of T cell receptors and flow cytometry excluded monoclonality. Based on the presence of EBV-LPD in a patient with personal history of microcephaly and psychomotor retardation a presumptive diagnosis of primary immunodeficiency with inability to control EBV was made. He was treated with rituximab for B lymphocyte depletion with resolution of fever and of the gastrointestinal bleeding, which were accompanied by marked decrease in the EBV viral load (viral load 50,000> 1015 copies/mL (**Figure 1D**). Hematopoietic stem cell transplantation (HSCT) was not proposed due to the personal history of severe psychomotor retardation.

Based on the pathologic findings of EBV+ T-cell LPD, and despite the absence of documented T-cell infection in the peripheral blood (5), cyclophosphamide was added to rituximab when the patient eventually presented a disease relapse, with fever, increasing viral load and generalized lymphadenopathies. This led to a dramatic clinical improvement and a decrease in viral load to 20 copies/ml (**Figure 1D**). Unfortunately, in the following months, the patient had several infectious episodes (namely central line related sepsis), leading to intermittent discontinuation of the immunosuppressive treatment and subsequent relapse of the disease with massive lymphoproliferation (**Figure 1C**) and increasing viral loads, eventually leading to his death.

A next-generation sequencing panel of EBV-susceptibility related genes (**Table S1**) led to the identification of a novel c.301G>A (p.(Ala101Thr)) homozygous ZBTB24 (NM_014797.2) mutation. This residue is highly conserved and there is a physicochemical difference between alanine and threonine (**Table S2**). This is a very rare variant and no homozygotes have been reported (total allele frequency of around 0.017%, 48 heterozygous; 0 homozygous). Most bioinformatics analysis predicted that the patient’s mutation Ala101Thr was damaging (e.g., SIFT = deleterious; MutationTaster = disease causing), with a CADD score of 17,49. This led to the diagnosis of immunodeficiency, centromeric region instability and facial anomalies syndrome (ICF) type 2. Both parents were found to harbor a heterozygotic mutation which allowed family counseling. Unfortunately, the patient died before karyotyping could be performed. We performed post-mortem Southern blot analysis for SAT2/Alpha-Satellite repeat methylation but did not find the characteristic ICF-associated repeat DNA hypomethylation. This is probably explained by the unusual nature of the mutation: a homozygous missense variant in the BTB domain.

## Discussion

ICF is a rare autosomal recessive disorder caused by defective DNA methylation, with approximately 70 cases reported so far (4). Hypomethylation of satellite regions on chromosomes 1, 9, and 16 and subsequent pericentromeric instability leads to chromosomal instability *in vitro*. Like our patient, individuals with this syndrome present facial anomalies such as ocular hypertelorism, epicanthic folds, broad flat nasal bridge, low-set ears, and macroglossia. Other traits of the disease comprise neurodevelopment disorder, including motor delay, speech delay, and intellectual disability (6). Moreover, patients with ICF have an impaired humoral and cellular immunity (4), leading to recurrent bacterial infections. Viral infections are less common but can have a severe course.

So far, four types of ICF syndromes have been identified. ICF1 is caused by mutations in DNMT3B, which encodes the DNA methyltransferase 3B and is the most frequent type of ICF syndrome, accounting for more than half of patients. ICF2 accounts for approximately 30% of ICF patients and is caused by mutations in ZBTB24, which encodes the zinc-finger-and BTB-domain (6). The remaining patients have mutations in CDCA7 (ICF3) or HELLS (ICF4) (7). Immunological findings appear to be similar in ICF1 and ICF2, though humoral immunodeficiency has been reported to be more severe in ICF1 still intellectual disability seems to be greater in patients with ICF2 (6).

ZBTB24 belongs to family of transcription factors, which form homo- or heterodimers in the nucleus *via* their BTB domain and bind to target genes *via* their DNA-binding C2H2 zinc-finger domains (6). ZBTB24 is highly expressed by B-cells and downregulation seems to increase the expression of IRF-4 (interferon regulatory factor 4) and Blimp-1 (B lymphocyte-induced maturation protein 1), two crucial factors involved in the proliferation and differentiation of B cell. These lock the cell-cycle in the G0/1- to S-phase without apoptosis induction (8).

Unlike our patient, that never had hypogammaglobulinemia, most (but not all) of the 40 patients reported with ICF2 presented hypogammaglobulinemia (particularly IgM) (6, 9). On the other hand, he had B cell lymphopenia with particularly low counts of post-germinal B cells, similar to what has been previously reported in these patients (9). Interestingly, five other ICF2 patients have been reported with EBV infection: the first was described as having “severe mononucleosis” (9), the second had persistent EBV infection (10), the third had an EBV-induced hemophagocytic lymphohystiocytosis (11), the fourth had a chronic EBV infection and developed an aggressive Hodgkin lymphoma (4) and the last one had an EBV-driven lymphoproliferative disorder with features of a CD20-negative large B-cell lymphoma (12) **(Table S3)** This susceptibility to EBV seems to be particular of ICF-2 as it has not been described in the other types of ICF. Similar to our patient, one patient had EBV+ T-cell lymphoproliferation (9) and other had progression of the disease despite rituximab (10). Knowing that EBV promotes epigenetic changes by changing methylation patterns (13) and that ICF patients present a pericentromeric repeat hypomethylation (14), it is possible to assume that differences in methylation profiles between ICF2 and the other types of this syndrome helps to explain their unique susceptibility to EBV (4).

Finally, it is mandatory to raise the hypothesis of an underlying PID in case of severe EBV infection, as HSCT is the only curative treatment in many of these cases. When HSCT is not an available therapeutic option (as in this case) the approach to chronic EBV infection is extraordinarily complex and should be individualized. The experience in controlling EBV in immunosuppressed patients is based on post-transplant lymphoproliferative disorders (PTLD) experience, where the mainstay is reduction of immunosuppression, which is an impossibility in primary immunodeficiency recipients. When B cell depletion with rituximab does not lead to a long-lasting control of the disease, regimens used in lymphoma therapy, such as cyclophosphamide, vincristine and prednisone, have been used despite an unsatisfactory outcome in many of these patients (15).

In summary, we report a patient who succumbed to EBV-T-cell LPD that harbored a novel homozygous A101T mutation in the ZBTB24 gene, causing ICF2. Future studies [such as the luciferase assay described by Daxinger et al (16)] will hopefully help us describe how this mutation affects ZBTB24’s role in controlling expression of CDCA7. This adds to the 5 previously reported cases in the literature, and we discuss the complex approach to the treatment of these patients in the absence of putative curative treatment.

## Data Availability Statement

The original contributions presented in the study are included in the article/**Supplementary Material**. Further inquiries can be directed to the corresponding author.

## Ethics Statement

Written informed consent was obtained from the minor(s)’ legal guardian/next of kin for the publication of any potentially identifiable images or data included in this article.

## Author Contributions

GP designed the study and wrote the paper. CA performed pathology analysis; AIC performed bibliographical research and was medical doctor of the patient; JF performed genetic analysis and reviewed the manuscript; CGM performed immunological analysis. JFN designed the study and analyzed the data. All authors contributed to the article and approved the submitted version.

## Funding

The present publication was funded by Fundação Ciência e Tecnologia, IP national support through CHRC (UIDP/04923/2020).

## Conflict of Interest

The authors declare that the research was conducted in the absence of any commercial or financial relationships that could be construed as a potential conflict of interest.
