# Supplementary material for: Case Report: Primary Immunodeficiencies, Massive EBV+ T-Cell Lympoproliferation Leading to the Diagnosis of ICF2 Syndrome
Source: Front Immunol. 2021 Apr 28;12:654167. doi: 10.3389/fimmu.2021.654167 (PMC8113761; doi:10.3389/fimmu.2021.654167)
Supplement: Supplementary file 1 [file DataSheet_1.docx]

**Table S1- Next-generation sequencing panel of EBV-susceptibility related genes**

ACD, ADA, AIRE, AK2, AP3B1, AP3D1, ARPC1B, ATM, B2M, BACH2, BCL10, BCL11B, BLM, CARD11, CARMIL2, CASP10, CASP8, CCBE1, CD247, CD27, CD3D, CD3E, CD3G, CD40, CD40LG, CD70, CD8A, CDCA7, CHD7, CIITA, CORO1A, CTC1, CTLA4, CTPS1, DCLRE1B, DCLRE1C, DKC1, DNMT3B, DOCK2, DOCK8, EPG5, ERCC6L2, EXTL3, FAAP24, FADD, FAS, FASLG, FOXN1, FOXP3, GINS1, HELLS, ICOS, IKBKB, IKBKG, IL10, IL10RA, IL10RB, IL21, IL21R, IL2RA, IL2RG, IL7R, ITCH, ITK, JAK1, JAK3, KDM6A, KMT2D, LAT, LCK, LIG1, LIG4, LRBA, LYST, MAGT1, MALT1, MAP3K14, MCM4, MSN, MTHFD1, MYSM1, NBN, NFAT5, NFKBIA, NHEJ1, NHP2, NOP10, NSMCE3, ORAI1, PARN, PEPD, PGM3, PMS2, PNP, POLE, POLE2, PRF1, PRKCD, PRKDC, PTPRC, RAB27A, RAG1, RAG2, RASGRP1, RBCK1, RELB, RFX5, RFXANK, RFXAP, RHOH, RMRP, RNF168, RNF31, RNU4ATAC, RTEL1, SAMD9, SAMD9L, SEMA3E, SH2D1A, SLC46A1, SMARCAL1, SP110, SPINK5, STAT3, STAT5B, STIM1, STK4, STN1, STX11, STXBP2, TAP1, TAP2, TAPBP, TBX1, TCN2, TERC, TERT, TFRC, TINF2, TNFRSF4, TPP2, TRAC, TTC7A, UNC119, UNC13D, WAS, WIPF1, WRAP53, XIAP, ZAP70, ZBTB24

**Table S2**

**Analysis of the variant found in ZBTB24**


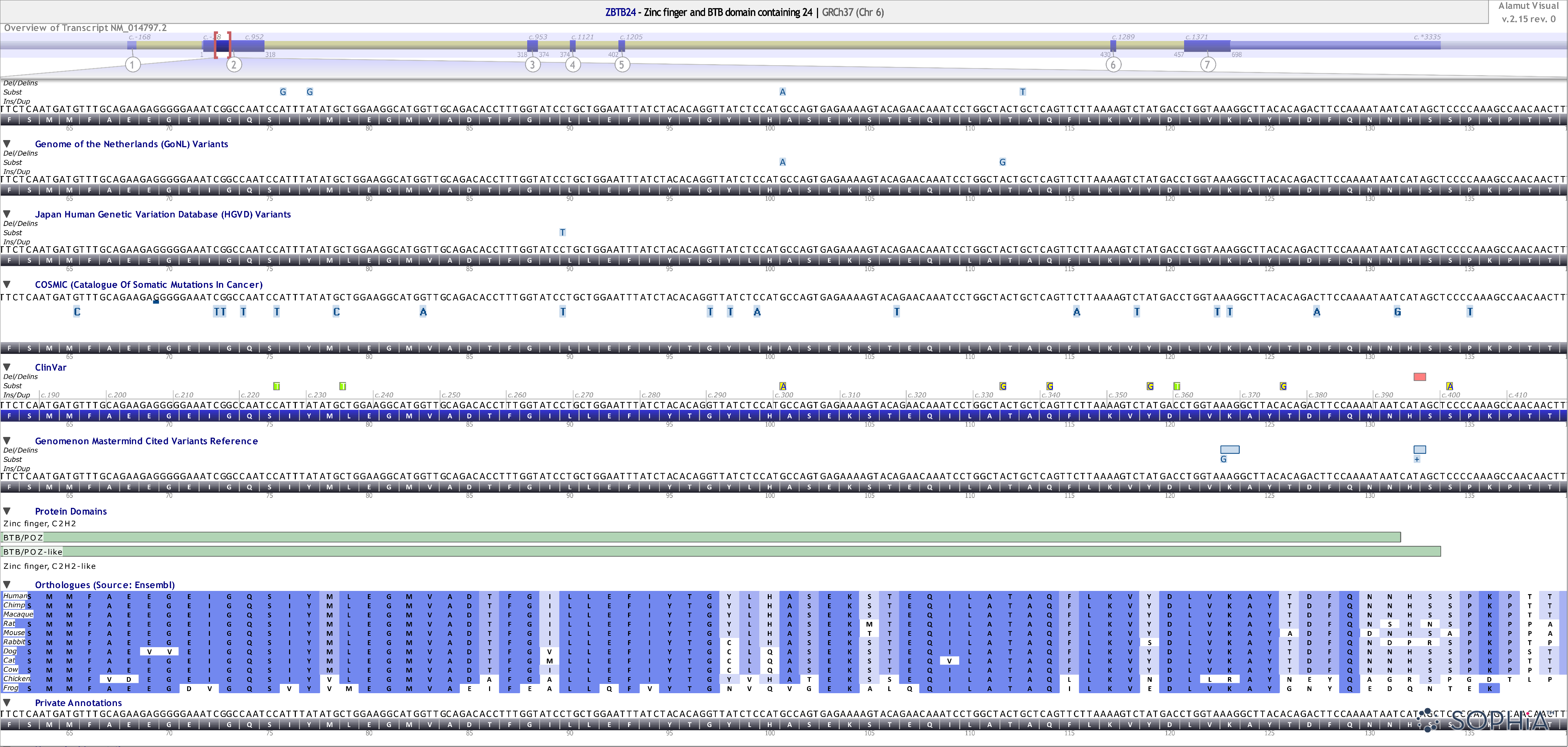

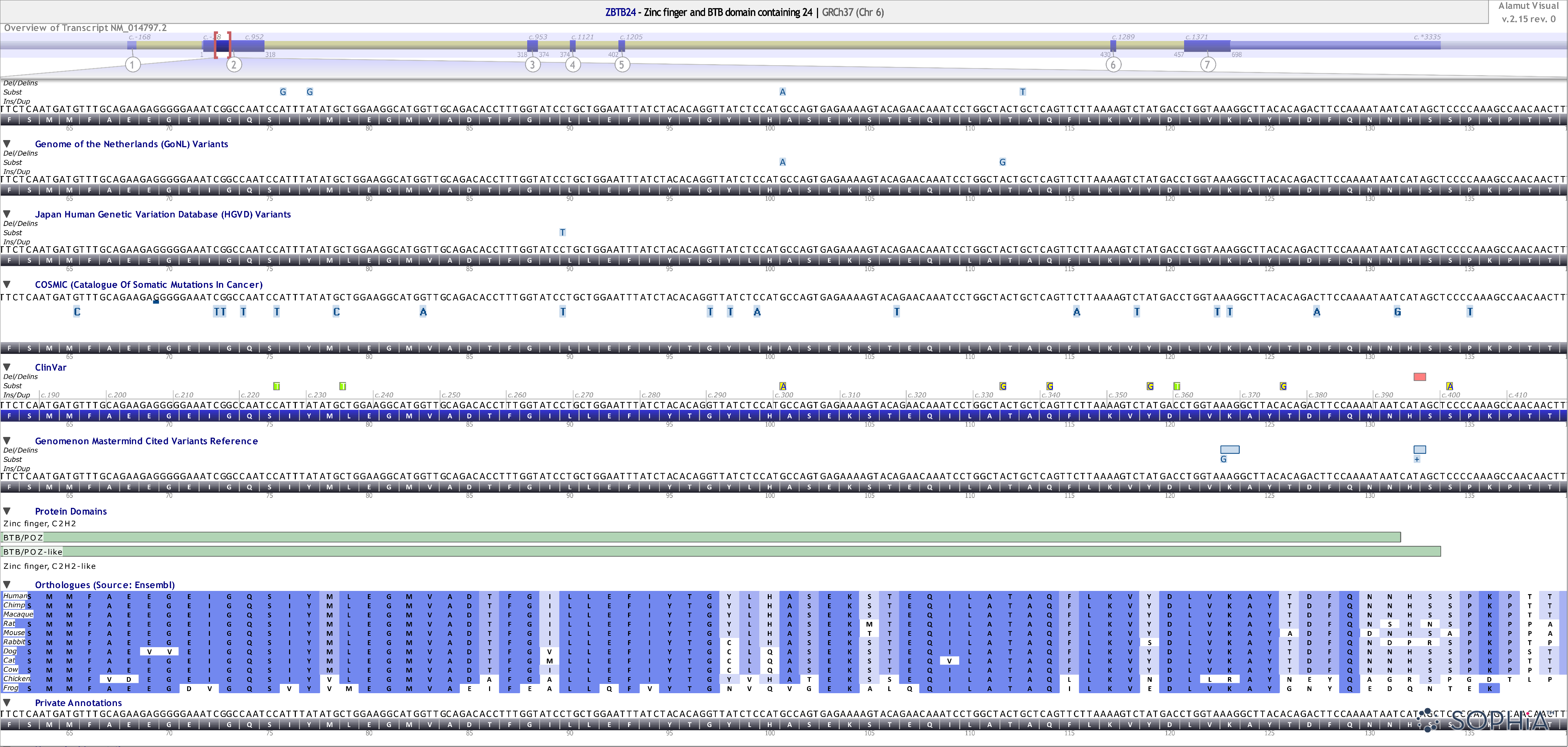


**T**

**Table S3**

ICF2-EBV patients

| **Identification** | **Mutation** | **Protein** | **Sex** | **Ethnicity** | **Facial anomalies** | **Intellectual development** | **Immunological findings** | **EBV manifestation** | **Outcome** | **References** |
| --- | --- | --- | --- | --- | --- | --- | --- | --- | --- | --- |
| **P1** | c.[980_981delGT];[787A>T] | p.[(Cys327Trpfs*54)];[(Lys263*)] | Fem | Cape Verdean | epicanthic folds | IQ<70 | low 27^+^ B cells, low IgM, no vaccine response to pneumococcus/ poliovirus/ diftheria/ tetanus toxoid, no IgM isoheamaglutinins, normal proliferation to PHA and tetanus and low to candida, NK cell count normal | severe mononucleosis | Stable (Immunoglobulin  +  Cotrimoxazole  +  Azythromycin | Nitta et al. 2013,  Sterlin et al. 2016 |
| **P2** | c.[1148G>A];[1148G>A] | p.[(Cys383Tyr)];[(Cys383Tyr)] | Mas | Japanese | macrocephaly, hypertelorism, epicanthalfolds, midface flatness, low nasal root, long flat philtrum, thick lips (upper lips<lower lips), genu valgum, irregular toes, hypoplastic primary teeth | normal | low CD27^+^ B cells, low IgM and low IgG2, decreased NK cell activity, no responses to vaccines | persistent EBV infection  EBV+ T-cell lymphoproliferation | Dead  (Immunoglobulin replacement therapy + trimethoprim–sulfamethoxazole | Nitta et al. 2013,  Kamae et al. 2018 |
| **P3** | c.[958C>T];[958C>T] | p.[(Arg320*)];[(Arg320*)] | Mas | Turkish | hypertelorism, broad nasal bridge, long philtrum, small low-set ears | IQ 49 | low IgM and low IgG | EBV-induced hemophagocytic lymphohistiocytosis | Favorable outcome (Dexamethasone, Etoposide, Cyclosporine  plus allogeneic hematopoietic  stem cell transplantation (HSCT)) | Harnisch et al. 2016 |
| **P4** | c.[175A>G];[175A>G] | p.[(Ser59Gly)];[(Ser59Gly)] | Mas | Italian | dolichocephaly, high forehead, hypertelorism/telecanthus |  | low IgG and IgM, low CD19^+^ cells, relative increase in increased CD8^+^ T cell count | chronic EBV infection with an aggressive Hodgkin lymphoma | complete  remission (doxorubicin +  bleomycin + vinblastine + dacarbazine (ABVD) for 6 cycles  plus rituximab (375 mg/m2)) | Licciardi et al. 2019 |
| **P5** | c.1492_1493del | p.Q498Vfs | Fem | Saudi arabia | hypotonia, arched eyebrows with blepharophimosis, telecanthus, ptosis, saddle-shaped  nose, recessed chin, | gross motor and speech delays at age 17  months. |  | EBV-driven lymphoproliferative disorder with  features of a CD20-negative large B-cell lymphoma | Favorable outcome with hydroxyurea, alemtuzumab, fludarabine, melphalan, and thiotepa followed by CD34-selected, 12/12 HLA-matched, unrelated-donor peripheral blood HSCT. | Burk CM, et al. J 2020 |
| **P6** |  |  | Mas | Portuguese | microcephaly, hypertelorism, low-set ears, epicanthal folds, and tongue hypertrophy | severe psychomotor retardation | Normal IG, severe T, B and NK-cell lymphopenia with particularly low counts of post-germinal B cells | persistent EBV infection  EBV+ T-cell lymphoproliferation | Death (Rituximab+ Cyclophosphamide +  Vincristine) | This paper |
