# Supplementary figures and images for: Case Report: Primary Immunodeficiencies, Massive EBV+ T-Cell Lympoproliferation Leading to the Diagnosis of ICF2 Syndrome
Source: Front Immunol. 2021 Apr 28;12:654167. doi: 10.3389/fimmu.2021.654167 (PMC8113761; doi:10.3389/fimmu.2021.654167)

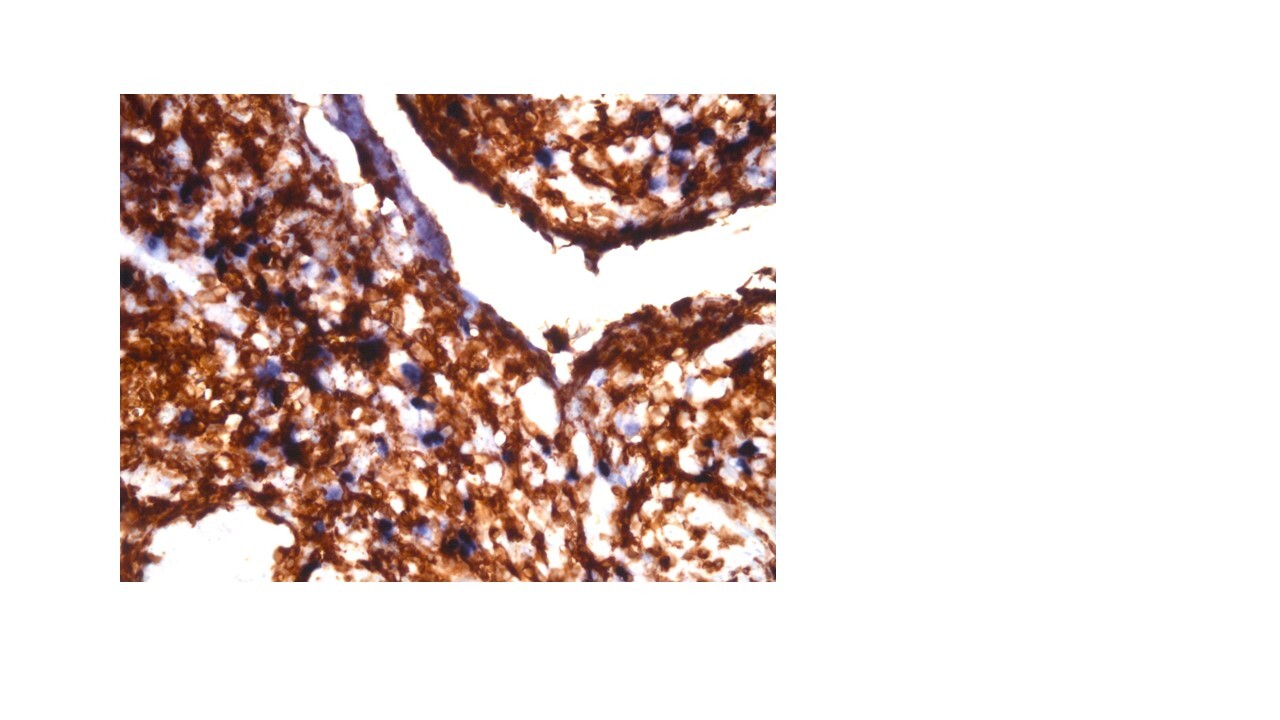

Supplement: Supplementary file 2 [file Image_1.jpeg]

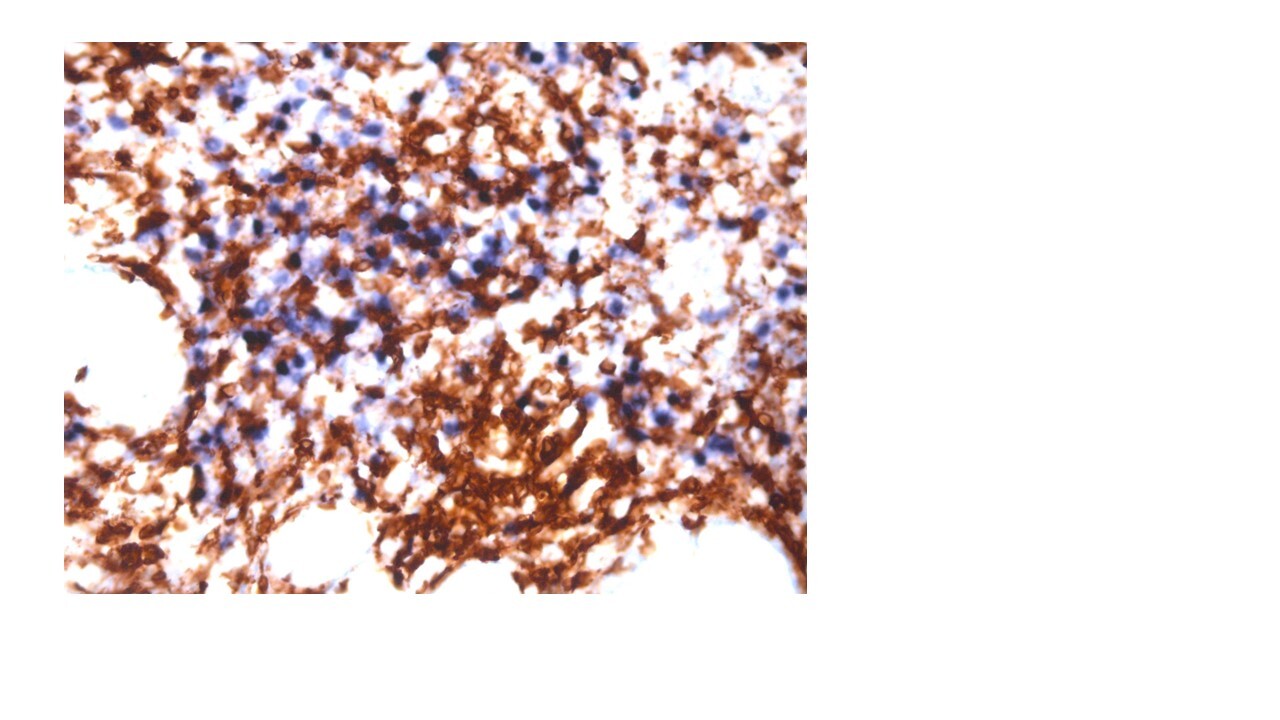

Supplement: Supplementary file 3 [file Image_2.jpeg]

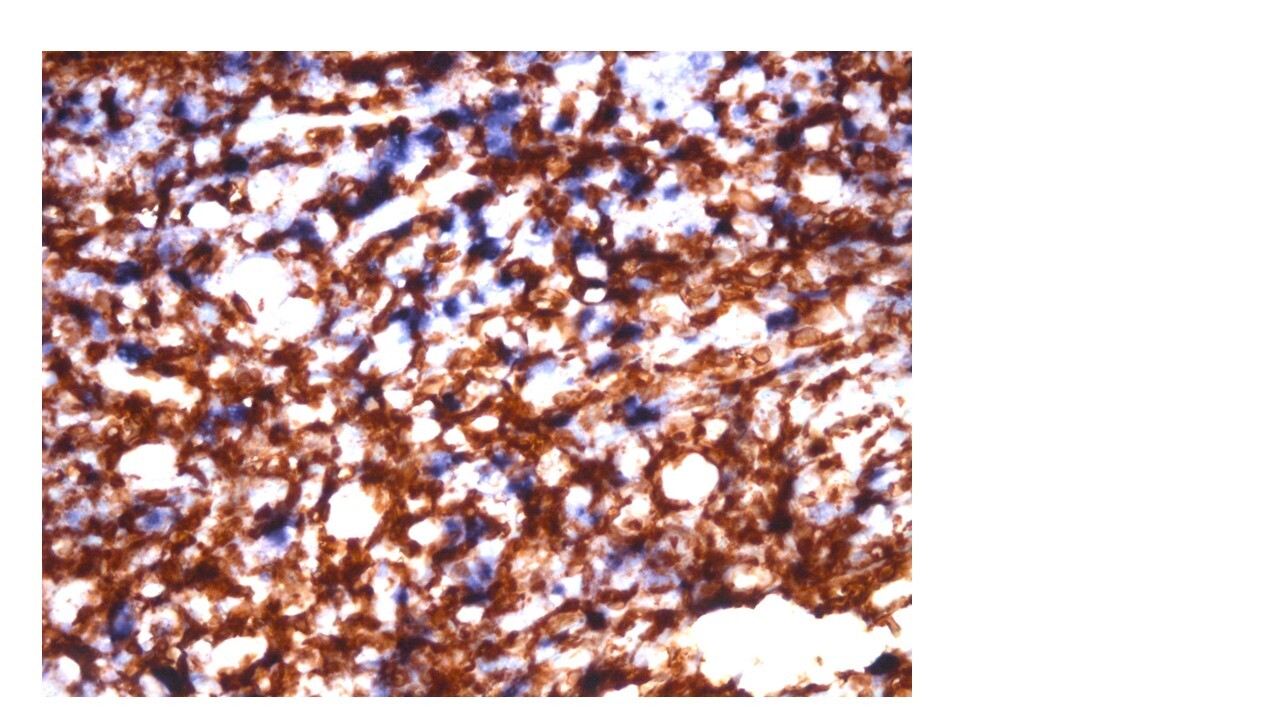

Supplement: Supplementary file 4 [file Image_3.jpeg]

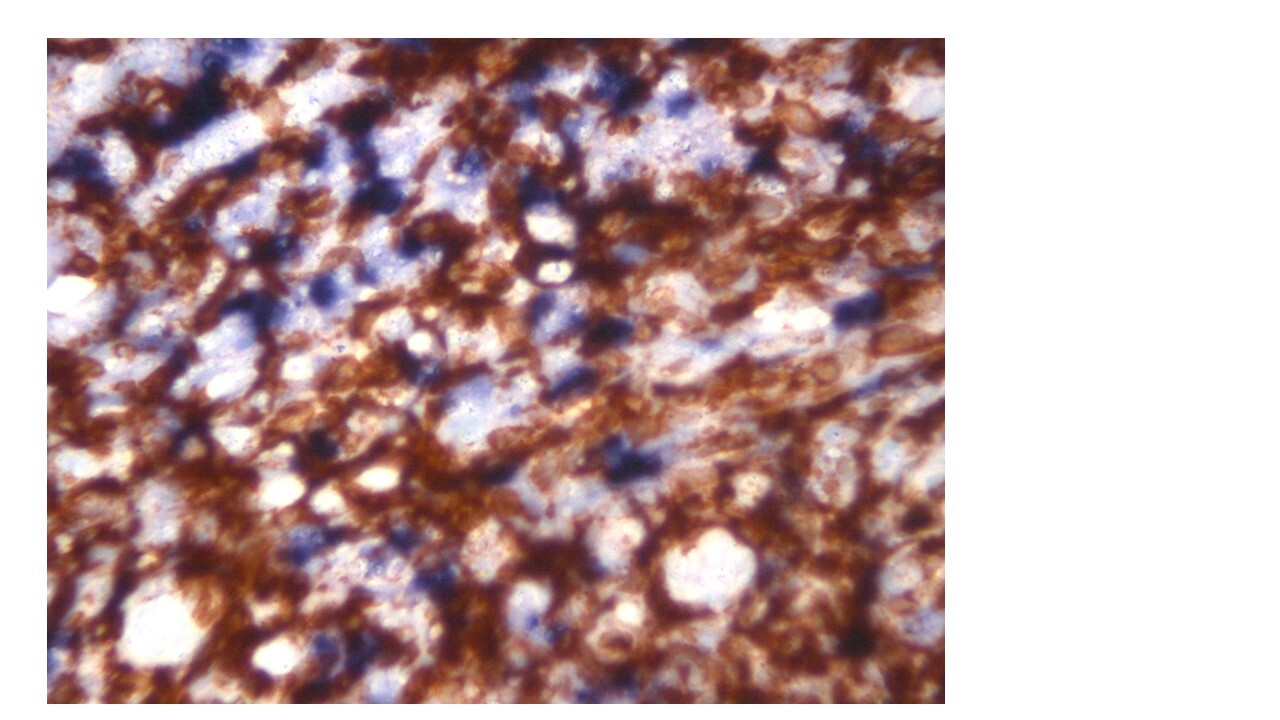

Supplement: Supplementary file 5 [file Image_4.jpeg]

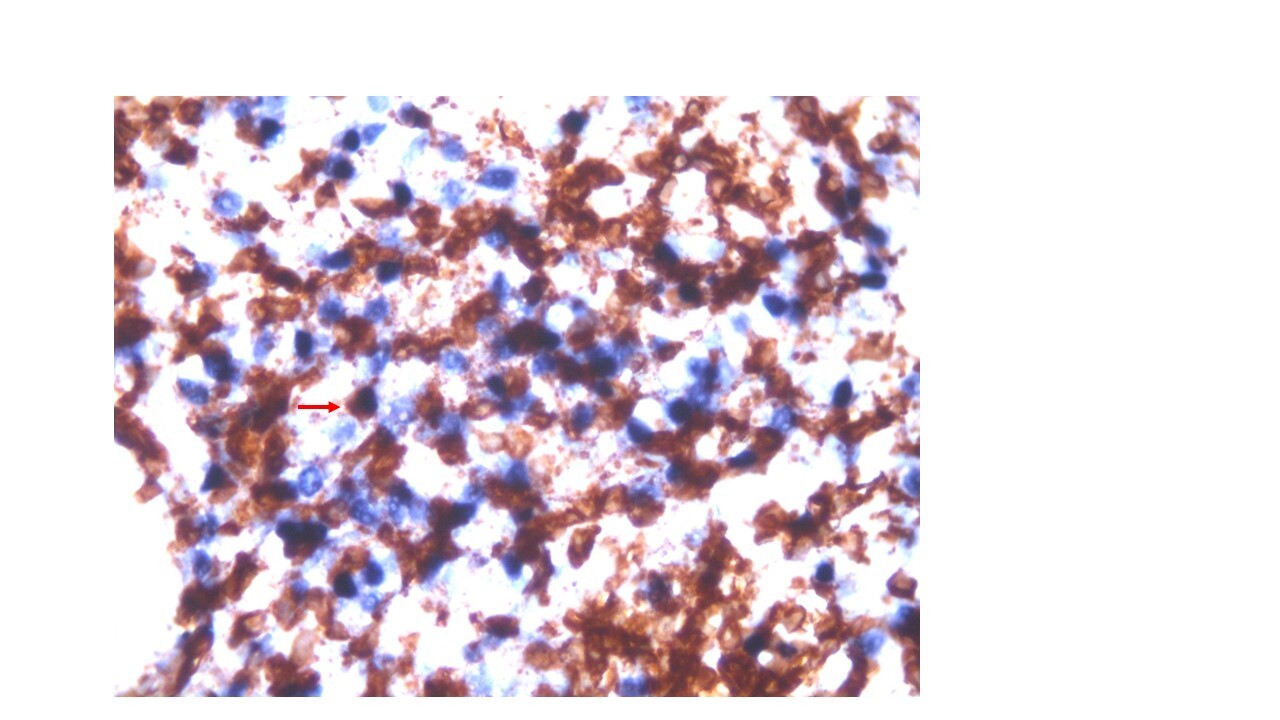

Supplement: Supplementary file 6 [file Image_5.jpeg]

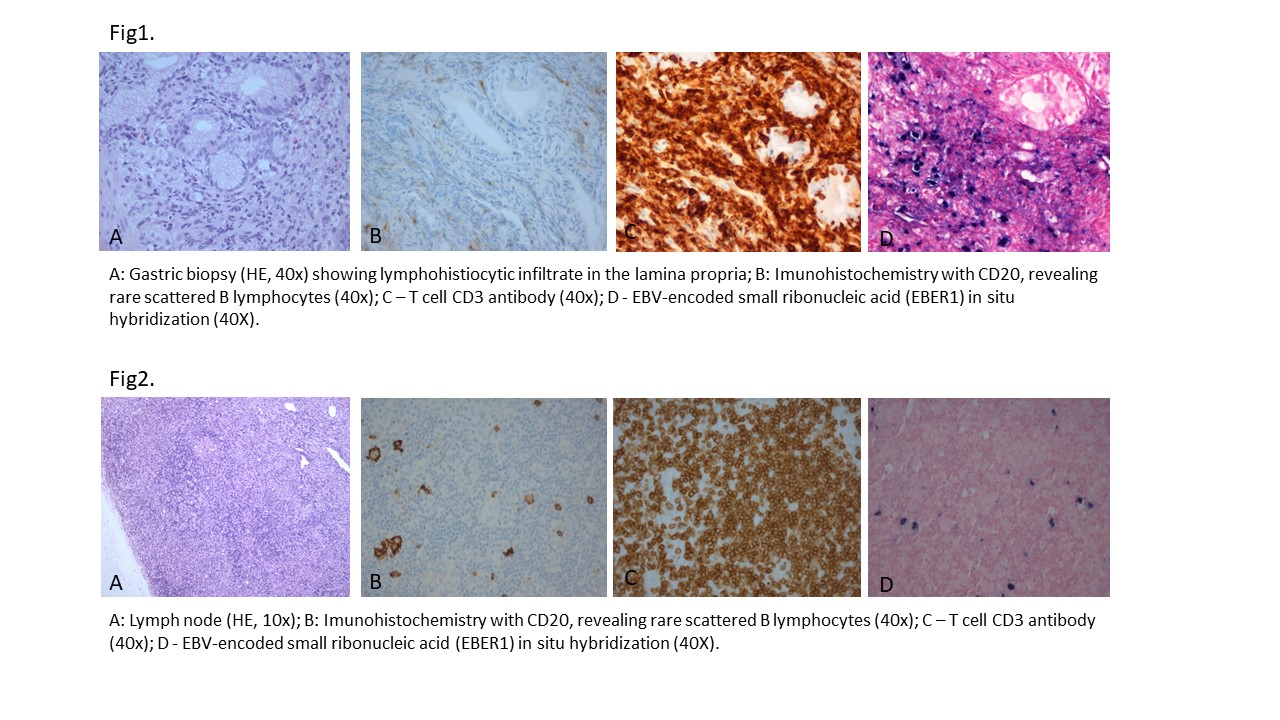

Supplement: Supplementary file 7 [file Image_6.jpeg]

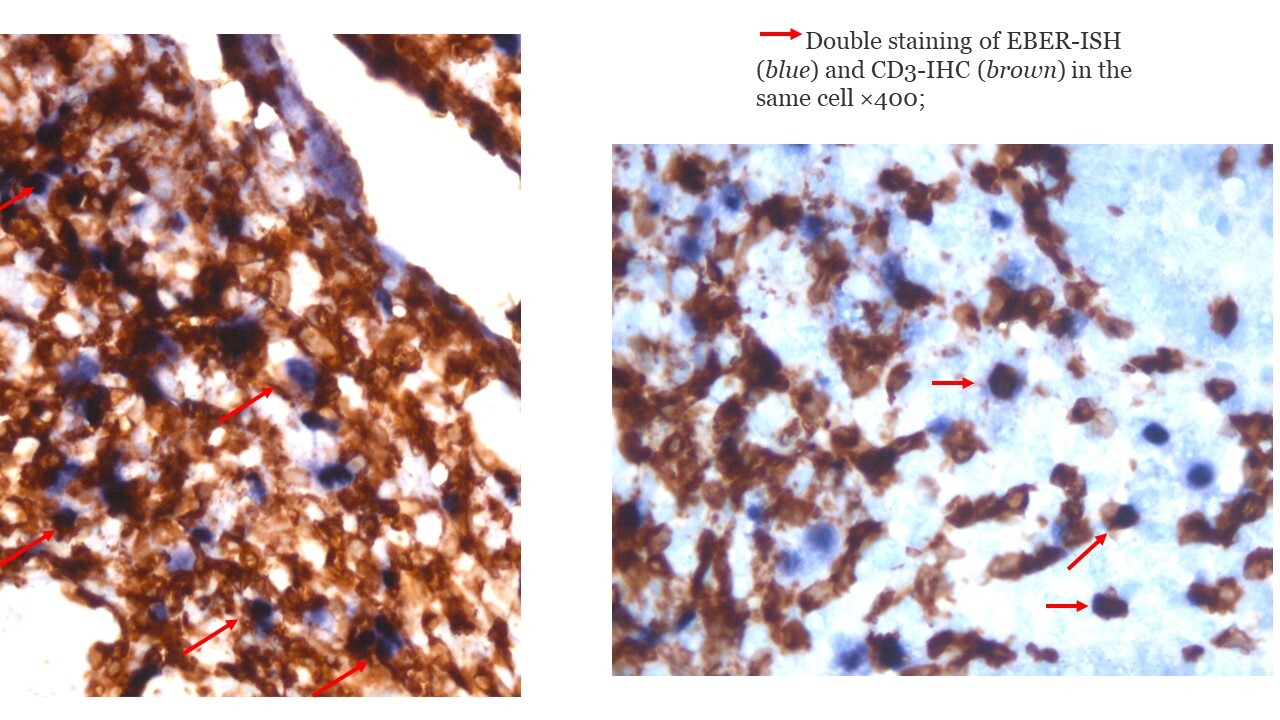

Supplement: Supplementary file 8 [file Image_7.jpeg]
